# The Impact of Genetic and Non-Genetic Factors on Lamb Loin Shear Force

**DOI:** 10.3390/ani14182628

**Published:** 2024-09-10

**Authors:** Hussein Al-Moadhen, Jarrod C. Lees, Julius H. J. van der Werf, Peter McGilchrist

**Affiliations:** 1 School of Environmental and Rural Science, University of New England, Armidale, NSW 2351, Australia; halmoad2@une.edu.au (H.A.-M.); j.lees@uq.edu.au (J.C.L.); jvanderw@une.edu.au (J.H.J.v.d.W.); 2 School of Agriculture and Food Science, The University of Queensland, Gatton, QLD 4343, Australia

**Keywords:** cold shortening, lamb, pH decline, shear force

## Abstract

**Simple Summary:**

Meat tenderness is crucial for eating quality, particularly in Australia, where it is a top palatability trait, with 50% consumer acceptance when shear force is below 42.6 N. Consumers are willing to pay a premium for guaranteed meat quality, making tenderness a priority for industry producers. Research has focused on the genetic and environmental factors affecting meat’s biological, structural and physiological characteristics. Objective evaluation methods like shear force are commonly used but can be influenced by pH–temperature interactions. Our findings indicate that controlling pH–temperature decline during processing enhances tenderness. Additionally, genetics can impact the risk of cold shortening.

**Abstract:**

Shear force is commonly used to evaluate tenderness, one of the most crucial eating quality aspects of sheep meat. The effect size of various factors on tenderness is still unknown. Studies have suggested that both genetic and environmental factors contribute to the variation in meat tenderness, and there are possible interactions between these factors. An extensive data set (n = 23,696) was analyzed to examine genetic and non-genetic influences on the shear force at 5 days postmortem (SF5). SF5 was measured on lamb loins (*Longissimus lumborum*) taken from lambs reared over 12 years at eight sites across Australia. The results showed that all carcass traits had a significant (*p* < 0.001) impact on SF5, with the largest effect on SF5 associated with intramuscular fat (IMF %) (*f* = 1035). There was also a significant effect of sex, cold shortening at 18 °C, sire type and cohort on SF5 (*p* < 0.001), with a large variation observed between the minimum cohort at 15.9 ± 1.5 N and maximum at 51.2 ± 2.1 N. In conclusion, a complex matrix of production, processing and genetic factors impact lamb tenderness as measured by shear force. This experiment helps identify the size of the contribution of these factors towards lamb tenderness, enabling the sheep industry to enhance consumers’ satisfaction.

## 1. Introduction

Tenderness of meat is critical to the eating experience [1,2,3] and is considered the most important qualitative characteristic of meat. Meat tenderness acceptability varies between countries [4,5] and species [6], but consumers of beef in Australia usually rate tenderness as the most important palatability trait, with 50% acceptance when shear force value < 42.6 [7]. Tenderness is a priority issue for the meat industry [8] and according to Lyford et al. [9], consumers are willing to pay a higher price for beef as long as it is guaranteed to be tender, highlighting the importance of meat tenderness in consumer acceptability. In addition, consumers of beef usually place the highest weight on tenderness and overall liking, followed by flavor and juiciness [10,11]. In lamb, tenderness is a highly variable characteristic, impacted by many intrinsic and extrinsic factors and their interactions. These factors are related to the animal (genetic and phenotypic traits), its management and the processing environment.

Given the relative importance of tenderness in meat quality, researchers have investigated how genetic and environmental factors affect meat biological, structural and physiological mechanisms and the relationship with tenderness [3,12,13]. The amount and solubility of connective tissue, sarcomere shortening during the onset of rigor linked to the pH–temperature decline, and postmortem proteolysis of myofibrillar and myofibrillar-associated proteins are all factors that impact the tenderness of meat [14]. Previous research has found that optimal lamb tenderness occurs when carcass pH reaches 6 when carcass temperature is 18 °C to 35 °C [15]. This pH–temperature combination is often referred to as ideal pH decline to minimize shortening and has been a focus of the Australian lamb meat industry to improve tenderness. Reaching pH6 at a temperature < 15 °C leads to cold shortening [16]. Fatter lamb carcasses are more insulated from rapid chilling so could be at lower risk of cold shortening.

Hocquette et al. [17] reported that intramuscular fat can also indirectly influence meat tenderness. However, the interaction between pH and fatness can be difficult to separate as each is influenced to a greater or lesser degree by genotype, pre-slaughter handling (including transport stress) and post-slaughter processes. Furthermore, these factors may have a relationship with a tenderness that is non-linear and dependent on muscle type and age of the animals [18].

Measurement of tenderness may be conducted by utilizing objective and/or subjective methods. Sensory testing using trained and untrained sensory panels may be used to predict meat eating quality, including meat tenderness [15,19]. Much of the work conducted on lamb eating quality in Australia has used untrained consumer sensory panels [20], where meat tenderness measurements are subject to individual consumer perceptions of desirable/undesirable tenderness. The wide variability in perception helps to govern what products are and are not acceptable, which is why consumers are used to establish meat value and build prediction models like Meat Standards Australia [2]. However, consumer sensory panels, both trained and untrained, are a high-cost method to assess meat tenderness [21] and are slow to generate data. Given the subjectivity of consumer scores, and the large number of samples required to account for the variation, a cheaper, more rapid and repeatable objective measure of tenderness is desirable [22] to make more rapid industry progress.

Tenderness can be evaluated by objective methods, such as shear force [2], and there have been attempts to devise instruments to assess the force required to shear, penetrate, bite, mince, compress or stretch the meat to yield a prediction of tenderness [3]. The most commonly used method for shear force measurement is Warner–Bratzler Shear Force (WBSF), which is a single-blade shear test that is pulled through cooked meat across the grain [23]. Caine et al. [24] reported that the correlations of WBSF with the sensory assessment of beef tenderness is variable, with R^2^ values ranging from 0.32 to 0.94. A study by Destefanis et al. [25] reported that the correlation coefficient of WBSF with sensory tenderness evaluation was −0.72, and a previous paper conducted on lamb meat identified that the tenderness prediction accuracy using shear force was low (R^2^ = 0.24; [26]). This variability in the relationship between measures could depend on many factors, including genetic and non-genetic effects, but, in general, the relationship between sensory and WBSF is moderate. Preliminary studies of lamb tenderness in Australia have utilized small numbers of animals over a few years [1,26,27,28]; however, large amounts of additional data were available for the present analysis. The objective of this experiment is to quantify the size of impact of production and processing factors along with carcass traits and genetics on the shear force of lamb loins as an indicator of tenderness.

## 2. Materials and Methods

### 2.1. Animals and Experiment Design

Research was conducted on lambs from the Meat and Livestock Australia resource flock and the Australian Sheep Co-operative Research Centre information nucleus flocks (INF). The design of the INF is detailed elsewhere [29,30]; however, briefly, 23,696 lambs were produced over a 12-year period at eight research sites across Australia. These 8 research stations were Kirby NSW (IN01), Trangie NSW (IN02), Cowra NSW (IN03), Rutherglen VIC (IN04), Hamilton VIC (IN05), Struan SA (IN06), Turretfield SA (IN07) and Katanning WA (IN08) (Table 1). These sites represent a broad cross-section of Australian sheep production systems. The lambs (Merino × Merino, Maternal × Merino, Terminal × Merino and Terminal × Border Leicester–Merino) were the progeny of 1229 key industry sires, representing the major production types in the Australian sheep industry. The sire types included Terminal sires (Hampshire Down, Ile De France, Poll Dorset, Southdown, Suffolk, Texel, White Suffolk); Maternal sires (Bond, Booroola, Border Leicester, Coopworth, Corriedale, Dohne Merino, East Friesian, Prime SAMM, White Dorper); and Merino sires (Merino, Poll Merino). Lamb birth date, birth type (single, twin, triplet or quadruplet) and weight were recorded within 12 h of birth [30] and a management ID tag was applied. Lambs were mainly maintained under pasture grazing conditions, but were fed grain, hay or feedlot pellets when pasture was limited [31]. Prior to slaughter, lambs were mustered, yarded, and taken off feed and water (from 2.5 to 18 h), allowing them to reduce gut fill prior to being weighed to predict dressing percentage. Lambs were transported to commercial abattoirs, where they were held in lairage overnight and slaughtered the following day. For each site, lambs were consigned to smaller groups which were killed on the same day (cohorts) to enable carcass weight targets to be achieved.

### 2.2. Animal Harvest and Chilling

The lambs were slaughtered between 134 to 766 days of age after head-only electrical stunning at a commercial abattoir. The carcasses were further electrically stimulated (800 mA with variable voltage to maintain a constant current, for 25 s at 14 pulses/s, 1 ms pulse width) post-dressing with a mid-voltage unit [32]. All carcasses were trimmed according to AUS-MEAT specifications [33]. Carcasses were chilled at a mean temperature of 3–4 °C over a 24 h period.

### 2.3. Sample Collection and Measurement

Hot carcass weight (HCWT) was measured after slaughter along with rib fat depth (11 cm from the midline to the lateral surface of the 12th rib, commonly called GR tissue depth) (HGRFAT) [23].

After the commencement of chilling, pH and temperature declines were measured in the left-hand portion of the *Longissimus lumborum* (LL) muscle at the caudal end over the lumbar–sacral junction. A section of subcutaneous fat and the *Gluteus medius* muscle was cut away to expose the LL; after measurement, the area was resealed with the overlaying tissue. The pH/temperature decline was measured until loin pH < 6.0 or carcasses were <12 °C. The pH meter was calibrated for temperature at 2 °C, which aligns with the chiller temperature, and calibrated for pH before use and every 2 h using pH 4.00 and pH 6.88 buffers at room temperature, as per Pearce et al. [32]. pH was first measured on entry to the chillers using meters with temperature compensation (WP-80, TPS Pty Ltd., Brisbane, Australia) and a polypropylene spear-type gel electrode (Ionode IJ 44), calibrated at ambient temperature. The second measure was captured after the temperature had dropped to around 18 °C or below; the third measurement was taken when the carcass was at roughly 12 °C. The pH of LL at 24 h postmortem (LL24pH) was measured in the caudal site used for repeat measures.

At 24 h postmortem, a 1-rib short loin (AUS-Meat code 4880 [34]) containing the LL muscle was excised from each carcass. At the cranial end or on the cut surface between the 12th and 13th rib, C-site fat depth (CFAT) and carcass eye muscle depth (CEMD) were measured. C-site fat depth is the depth of fat over the LL, measured using calipers or a ruler, 45 mm from the back line. CEMD is the depth of the LL, also measured 45 mm from the back line, from the dorsal to ventral edges of the LL.

For determination of IMF%, approximately 40 g of diced loin muscle was collected in 50 mL tubes from the cranial end of the LL. Samples had a wet weight recorded and then were stored at −20 °C until subsequent freeze drying. Samples were commercially freeze-dried using a Cuddon FD 1015 freeze dryer (Cuddon Freeze Dry, Blenheim 7201, New Zealand). To determine IMF% content, a near-infrared procedure (NIR) was used in a Technicon InfraAlyzer 450 (19 wavelengths) [23]. NIR readings were validated with chemical fat determinations using solvent extraction (chloroform in a soxhlet) and IMF% was expressed as a percentage of fat.

For shear force testing, samples of LL from the cranial end, behind where the IMF% sample was taken, were prepared into 65 g blocks after 24 h postmortem. Samples were then vacuum packed and held chilled (1 °C) until freezing on day 5 at −20 °C. Samples for shear testing were cooked from frozen for 35 min in vacuumed plastic bags at 71 °C in a water bath, as previously described by Hopkins and Thompson [34], before being tested using a Lloyd Texture Analyzer (Model LRX, Lloyd Instruments, Hampshire, UK) with a Warner–Bratzler type shear blade fitted.

### 2.4. Classification of Cold Shortening

The risk of cold shortening (CS) carcasses was analyzed using R [35]. To estimate the risk of CS, the model used the three pH–temperature decline readings (pH1, pHTEMP1, pH2, pHTEMP2, pH3, and pHTEMP3). A linear model was used to estimate the gradient and intercept of the pH–temperature decline for each individual carcass. In order to determine CS, a linear equation was used to estimate pH at different temperatures (18, 15, 12, 10 °C) for the available lambs (n = 21,547) where the linear fit was achieved. Carcasses were classified as normal—which is carcass pH = 6 (pH6) and temperature between 18–35 °C—or as CS—which is carcass pH greater than 6 and temperature below 18 °C—using binominal characters (0 = normal, 1 = CS).

### 2.5. Statistical Analysis

Of the total 23,696 lambs available for interrogation, complete data were available from only 18,024 lambs for the analysis. Shear force (SF5) was analyzed using a linear regression model (lm package) in R [35]. The model included cohort, sire type (Terminal, Maternal and Merino), sex (male and female), age at slaughter (days), birth type (single, twin, triplet or quadruplet) and cold shortening risk (0 or 1). Several phenotypic traits and their quadratic effect were fitted as covariates and were tested by adding each individually in the base model described above to determine their association with shear force and then all together. These traits were hot carcass weight (HCWT), C-site fat on cold carcass (CFAT), GR fat depth (GRFAT), carcass eye muscle depth (CEMD) and intramuscular fat (IMF%). All non-significant (*p* > 0.05) terms and first order interactions between fixed effects were removed in a stepwise manner.

## 3. Results

### 3.1. Animals

Of the total 23,696 animals with data available, the base models used 18,024 animals that had the full complement of production data. The raw data showed the average SF score for all available lambs was 32 N, with a maximum of 105 N and a minimum of 11 N. The first quartile and the third quartile were 24 and 37 N, respectively.

### 3.2. The Impact of Fixed Effects on Shear Force (SF5) at Day Five

**Figure 1:**
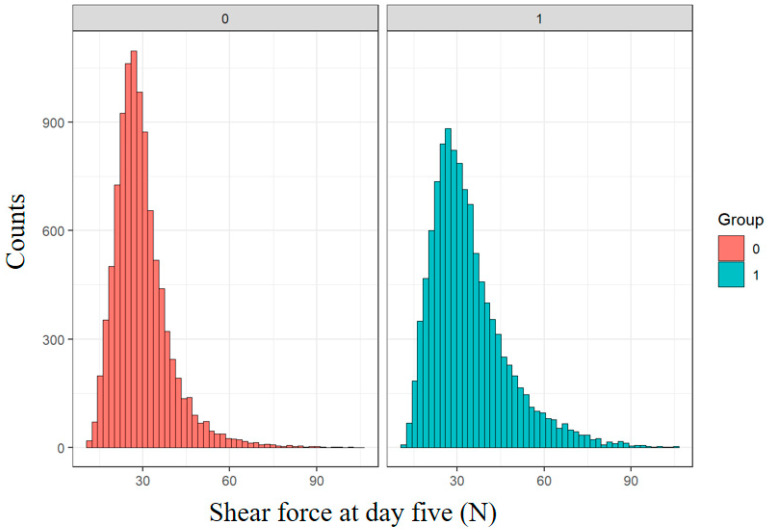
Cold shortening prediction model and the differences in shear force between normal = 0 and cold shortening = 1, carcasses at 18 °C.

The outcomes from the analysis models are presented in Table 2. The base model included the significant terms cohort, sire type, sex, age at slaughter and cold shortening at 18 °C, which explained 38% of variation in shear force. From the base model, cold shortening risk had the highest impact on SF5 (*F* = 433) and a consistent effect across all models even when carcass traits were added in to the base models (Figure 1 and Table 2). Figure 1 shows the SF5 distribution of lambs classified as CS (pH6Temp < 18 °C) against those that were not classified as CS. There is a much larger tail to the right of the CS group which is reflected in the average SF5 values of the two groups, which were 31.2 ± 0.14 N, compared to 27.4 ± 0.16 N for the non-CS lambs (Figure 1).

Likewise, there was a significant effect (*p* < 0.001) of gender on SF5, with males having a 5% higher mean SF5 (31.3 ± 0.66 N) than females (29.8 ± 0.66 N, *p* < 0.001). Cohort had a significant impact on SF5 (*p* < 0.001), and differences in the estimated mean SF5 value for cohorts ranged from 15.9 ± 1.5 to 51.2 ± 2.1 N.

Sire type also had significant impact on SF5 (*p* < 0.001, Table 2), with Merino sire progeny having lower SF (26.3 ± 0.21) than progeny of Maternal sires (27.1 ± 0.17) and those of Terminal sires (29.1 ± 0.12) by 7.7% and 13.9%, respectively (*p* < 0.001).

There was a significant linear effect of age at slaughter on SF5 (*p* < 0.001). As slaughter age increases from 150 days to 350 days, SF decreases by 4 N, from 31 N to 27 N, and this effect did not change even when carcass traits like IMF% were included in the model.

### 3.3. Phenotypic Effects on Shear Force (SF) at Day Five

**Figure 2:**
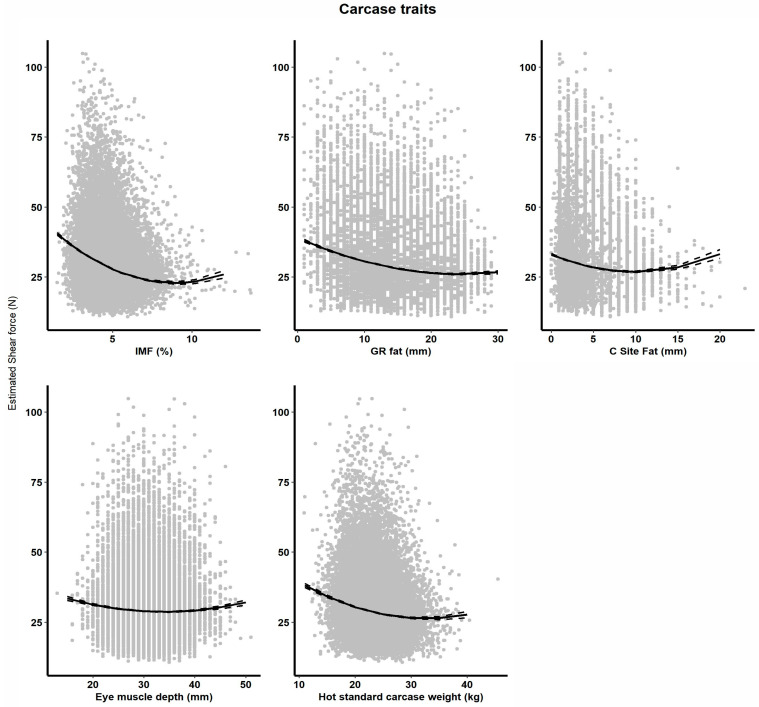
Estimated marginal mean (Emmeans) effects (±standard error) of phenotypic traits for hot standard carcass weight (kg), eye muscle depth (mm), GR fat depth (mm), C-site fat depth (mm) and intramuscular fat (%) on shear force (N) at day five.

When HCWT, CEMD, CFAT, GRFAT and IMF% are added to the base model as linear and quadratic covariates, the variance explained was 39%, 38%, 39%, 40% and 42%, respectively. All carcass trait covariates were significant (Table 2). However, IMF% had the largest impact on SF5 and was a consistent effect even when all carcass traits were tested in one model (*p* < 0.001, Table 2). Its inclusion in the model also explained 4% more variation in SF5 than the base model alone (38 vs. 42%). There was a significant (*p* = 0.01) negative effect of IMF% on SF5 value. Increasing IMF% from 2% to 14% decreased SF by 14.8 N. When the quadratic term is fitted in the model, the relationship is still linearly negative until IMF% becomes greater than 10%, where the effect plateaus and then increases, which is likely due to low data numbers (Figure 2).

Hot carcass weight had a significant (*p* = 0.01) negative effect on SF5 value. Increasing HCWT from 15 kg to 30 kg decreased SF5 by 7.7 N. There is a curve linear effect of HCWT on SF5 (*p* < 0.001) until the relationship plateaus at about 30 kg HCWT (Figure 2), above which there is minimal effect of HCWT on SF5.

There was a significant (*p* < 0.01) negative quadratic effect of GR fat and CFAT on SF5 value (*p* = 0.01). As GR fat increased from 5 to 30 mm, SF5 value decreased by 11.3 N (Figure 2). As CFAT increases from 5 to 10 mm, there is a 1.5 N decrease in SF5 and then the relationship reverses, showing a negative impact of CFAT on SF5 at ranges greater than 15 mm (Figure 2). The CEMD analysis showed a significant (*p* < 0.001) negative relationship with SF5. Increases in the CEMD from 10 mm to 30 mm decreased SF by 7.4 N. Due to the quadratic nature of the relationship between SF5 and CEMD, as CEMD increased beyond 35 mm, there was a slight positive relationship with SF5 (Figure 2).

## 4. Discussion

The present study had a large number of kill groups (195) and the associated large variation in SF5 is not surprising as environmental variables such as season, feed availability between the years, nutrition, and variation in handling during transport and processing are all possible contributors to variation. CS at 18 °C had the largest impact on SF5 in the base model, followed by gender and cohort; however, when carcass traits were included in the models, IMF% overtook CS risk as the largest contributor to variation in SF5, rejecting the initial hypothesis. CS is clearly an environmental effect at the processor. The number of animals used in this study clearly showed the significant effect of pH/temperature decline on CS and the resultant increase in SF5 and decrease in tenderness. From the size of the F-values in the statistical models shown in Table 2, cold shortening risk has the second largest impact on SF5, indicating that processing conditions are paramount in ensuring the tenderness of lamb produced. A rapid decline in temperature is known to induce ‘cold shortening’ of muscle fibers and to increase meat toughness [21,36,37]. Previous studies have used pH–temperature data to predict meat tenderness [16,21]; the present study used a similar parameter of pH decline within a set temperature range of 18 °C, with a large number of animals used to categorize cold shortened and ‘normal’ carcasses. There was difficulty in predicting cold shortening using pH–temperature decline data (Figure 1). This was primarily caused by some animals missing one or two records either for pH or temperature, which may have adversely affected the accuracy of the prediction model for temp at pH6. Furthermore, each carcass displays a different pH–temperature decline path due to extrinsic factors such as chilling rate, carcass fatness, position in the chiller, time of kill, season and concentration of glycogen [38]. Information was not available on the chilling environment; however, improvements in the prediction of cold shortening may be possible by adding environmental effects such as chain speed, chill temperature and chiller rate for each cohort. The models show an average 3 N difference between the normal and cold shortened carcass loin, which is around 50% of total variation in loin SF5, suggesting that more needs to be done to control pH decline in lamb carcasses or at least to avoid cold shortening. There is a strong relationship between sarcomere lengths and pH–temperature decline. Sarcomere lengths are essential to the determination of tenderness [39]. In cases where carcass temperature falls below 10–15 °C in the early postmortem period, calcium is released from the sarcoplasmic reticulum however glycolysis is still occurring so there is sufficient residual ATP available, allowing the muscle fibers to drastically shorten [40]. The postmortem glycolysis occurs until glycogen stores are depleted, resulting in lower pH [40,41]. Key factors that are likely to be associated with temperature decline include carcass weight, which affects muscle size; GR fat; C fat; and other carcass traits. For instance, the amount of fat surrounding the loin muscle could prevent rapid chilling of the muscle (chilling rate). Chill rate can also be affected by the position of the carcasses in the chiller, as the closer they are to the blowing air, the quicker they chill. Chain speed might affect how quickly the carcasses enter the chiller [36,37] and such factors can contribute to an increased risk of cold shortening. Previous studies [42,43,44] have shown that there are many physiological events that occur to convert muscles to meat, and the processor factors make the prediction model for lamb tenderness complicated.

Our results showed that an increase in IMF%, GRFAT, HCWT and CFAT was associated with lower shear force value for the loin sample, which is in agreement with other studies [2,3,17,26]. Interestingly, when all carcass traits were included in the model, IMF% had the largest positive impact on SF5. It well known that meat tenderness has been previously associated with IMF% score [15], and as IMF% and rib fat are positively related [8], it is not surprising that measures of increasing fat proportion are associated with a decrease in SF5 value across a diverse range of genotypes and environments. Pannier et al. [45] reported that, across the kill groups, there was a positive relationship between age at slaughter and IMF% score and that this relationship could affect shear force. Age at slaughter was significant in the base model; however, even when IMF% was included in the model, the F-value for age at slaughter did not change. This was the same when all carcass traits were added into the model, indicating that older lambs genuinely did have lower SF5 values. Increasing age by 200 days decreased SF5 by 4 N. An increase in age at slaughter was associated with lower shear force, which contrasts with some reports that have shown that consumers rate lamb meat from older animals tougher, which is reflective of increasing shear force value [46,47]. One possible explanation for this is that the differential in age seen in the lambs of this experiment was not large enough to generate differences in collagen content of the LL, thus not affecting SF5.

The current study showed mean SF5 values were higher for males by 1.6 N than females. This slight difference between males and females is in agreement with other studies [48,49,50]. Including sex in the analysis models was significant and did not differ when carcass traits were added to the models. Consistent differences in some aspects of carcass quality between male and female lambs have been reported in the literature, with carcasses from male lambs being heavier at the same age and having lower dressing out percentages and poorer conformation scores, even though they also have a lower proportion of fat and higher lean meat yields [50,51,52]. This could explain why females have more IMF%, which is in agreement with Pannier et al. [46], who showed that females deposit 0.15% more IMF% in the loin than males. This could be because males are growing faster than females and reach heavier weights at slaughter, which might lead to leaner meat and lower fat proportion [50,52,53]. This could change muscle fiber structure during growth [54]. There is evidence that muscle fiber [55], maturity stage [56] and muscle bundle size differences [57] between males and females affect muscle structure and SF5. All the above combined might be the reason for the differences in our results between males and females.

The present study used a large diversity of sire breed types, with the Terminal sire group showing the higher SF5 for the LL. This difference may be associated with greater intensity of selection for more muscular and leaner carcasses in order to improve meat yield in Terminal breeds. Increasing post-weaning weight (PWWT) ASBV is associated with increased slaughter weight and increasing eye muscle depth (PEMD) ASBV improved dressing percentage. Greater selection intensity of these two traits in Terminals could increase production advantage and profitability [58] but might have negative impact on meat tenderness [51]. Selection for higher lean meat yield (LMY) has been shown to increase toughness [46]. The biggest gains in LMY have been in the Terminal breeds. The mechanism driving high SF5 values in Terminal breeds is unclear; however, it could be assumed that it is related to the rate of proteolysis and reduced calpain activity in the early postmortem period [59]. It is possible that Terminal sires have more calpastatin in their muscles, which can increase meat toughness [60] via reduced proteolysis. Pomponio and Ertbjerg [59] also showed that the calpain system was affected by prolonged exposure to temperatures in the range of 2–30 °C. It is well established that postmortem proteolysis of myofibrillar and myofibrillar associated proteins by calcium-activated proteases results in lower shear force [61,62]. However, this study assumes that the relationship between the calpain system and postmortem tenderization between breeds may be more complex than initially thought.

The relationship between SF5 and carcass traits is not linear [18]. Within this research, it was found that a quadratic term explained a greater degree of the relationship between SF5 and HCWT, CEMD, CFAT and GR fat. An increase in CEMD has been documented to reduce IMF% [45]. IMF% is known as the biggest driver of SF5 [17]; hence, selecting for larger CEMD is not favorable for IMF%, which reflects on SF5. However, even when both are in the statistical model, both remain significant, which means they have related, but slightly different modes of action with respect to IMF%. The relationship between SF5 and CFAT shows a decrease in SF5 value as the fat mass increases. As discussed previously, increasing weight and fat depths may have reduced the lambs’ risk of cold shortening. Hence, heavier, fatter lambs could have lower SF5 values because these terms in the model may explain some variation in SF5 that the CS term did not explain. Animal fatness is, of course, affected by environmental factors such as feed availability and nutritive value. Hence, carcass traits might also indicate that animals with better nutrition have lower SF5. Animal nutrition and health was not available to add into the statistical models, so carcass traits may partially explain differences. The current study also had 195 cohorts, and the effect of the cohorts on SF5 was significant and consistent across all models, even when all carcass traits were included in the model. These cohorts ranged from 15.9 ± 1.5 to 51.2 ± 2.1 in mean SF5 values. This wide range in the SF5 value is due to many factors confounded and nested within cohorts, such as handling, seasons and processing factors like electrical stimulation and chill rates, which could differ slightly between cohorts.

## 5. Conclusions

This study confirms that pH–temperature declines in lambs have the second largest impact on SF. However, it was difficult to accurately predict, using pH decline data, which carcasses could be assigned as at a high risk of cold shortening due to the difficulty of accurately measuring pH and temperature in meat. This study demonstrated that carcass traits had a significant impact on SF. IMF% had the largest impact on SF across all the factors in the model, followed by HCWT and GR fatness. Sex and C-site fatness had the next largest effect, followed by cohort, sire type, eye muscle depth and age at slaughter. This study highlights that there is a complex matrix of factors that impact lamb tenderness as measured by shear force, but the largest contributors to variation are IMF% and cold shortening. These results can assist sheep producers and processors in identifying the size of contribution of various genetic, production and processing factors to lamb tenderness to ensure satisfaction of consumers.

## Figures and Tables

**Table 1:** Number of animals slaughtered across the 12 years from eight sites (IN01 to IN08).

	Flocks *								
Year	IN01	IN02	IN03	IN04	IN05	IN06	IN07	IN08	Total/Year
1	248		298	298	224	298	270	417	2053
2	400	303	158	309	195	132	280	580	2357
3	493	282	328	389	218	264	318	409	2701
4	370	267	197	208	197	183	299	462	2514
5	399	127	264	286	232	188	292	401	2386
6	1326	-	-	-	-	-	-	821	2147
7	1128	-	-	-	-	-	-	674	1802
8	975	-	-	-	-	-	-	855	1830
9	877	-	-	-	-	-	-	858	1735
10	719	-	-	-	-	-	-	542	1563
11	890	-	-	-	-	-	-	918	2007
12	709	-	-	-	-	-	-	821	1678
Total animals	8534	979	1245	1490	1066	1065	1459	7758	23,696

* Kirby NSW (IN01), Trangie NSW (IN02), Cowra NSW (IN03), Rutherglen VIC (IN04), Hamilton VIC (IN05), Struan SA (IN06), Turretfield SA (IN07) and Katanning WA (IN08).

**Table 2:** F-value and degree of freedom (DF) for the effects of the base linear effects models, corrected for hot standard carcass weight (HCWT), eye muscle depth (CEMD), C-site fat (CFAT), GR fat (GR FAT) and intramuscular fat% (IMF%) of lamb muscle.

	Base Model		Model Corrected for Hot Carcass Weight (kg)	Model Corrected for Eye Muscle Depth (mm)	Model Corrected for C-Site Fat (mm)	Model Corrected for gr Fat (mm)	Model Corrected for Intramuscular Fat %	Model Included All Carcass Traits	Model Included Carcass Traits and Quadratic Terms
EFFECT	DF	F-Value	F-Value	F-Value	F-Value	F-Value	F-Value	F-Value	F-Value
Cohort	195	53 ***	53 ***	54 ***	54 ***	55 ***	57 ***	55 ***	56 ***
Sire type	2	24 ***	24 ***	23 ***	23 ***	25 ***	25 ***	24 ***	24 ***
Gender	1	139 ***	144 ***	133 ***	134 ***	143 ***	147 ***	146 ***	148 ***
Age at slaughter	1	8 **	8 **	8 **	7 **	8 **	8 **	8 **	8 **
Cold shorten 18	1	433 ***	430 ***	432 ***	436 ***	444 ***	454 ***	453 ***	457 ***
HCWT	1	-	228 ***	-	-	-	-	234 ***	236 ***
HCWT^2^	1	-	40 ***	-	-	-	-	-	48 ***
CEMD	1	-	-	11 ***	-	-	-	7 **	10 **
CEMD^2^	1	-	-	28 ***	-	-	-	-	6 *
CFAT	1	-	-	-	210	-	-	101 ***	97 ***
CFAT^2^	1	-	-	-	67 ***	-	-	-	43 ***
GR FAT	1	-	-	-	-	490 ***	-	192 ***	164 ***
GR FAT^2^	1	-	-	-	-	94 ***	-	-	52 ***
IMF%	1	-	-	-	-	-	1035 ***	772 ***	729 ***
IMF%^2^	1	-	-	-	-	-	129 ***	-	88 ***

* *p*-value < 0.05, ** *p*-value < 0.01, *** *p*-value < 0.001.

## Data Availability

The original contributions presented in the study are included in the article, further inquiries can be directed to the corresponding author. None of the data were deposited in an official repository.
